# Music Intervention for older adults: Evidence Map of Systematic Reviews

**DOI:** 10.1097/MD.0000000000036016

**Published:** 2023-12-01

**Authors:** Guiyue Ma, Xiaoqin Ma

**Affiliations:** a School of Nursing, Anhui University of Chinese Medicine, Hefei, China; b School of Nursing, Zhejiang Chinese Medical University, Hangzhou, China.

**Keywords:** cognition, evidence map, music, psychology, systematic review

## Abstract

**Background:**

With the increasing aging population, the health problems of the elderly have received increasing attention. As a non-pharmacological interventions, music intervention has been widely used in clinical practice to improve the physical and mental health of the elderly. This article aims to provide a comprehensive review of existing systematic reviews on the health effects of music interventions for older adults in clinical practice.

**Methods:**

The study utilized the evidence map methodology, which involved identifying all relevant systematic reviews, meta-analysis from 7 electronic databases from their inception to November 2022. The studies were analyzed using AMSTAR 2.

**Results:**

The researchers identified 67 studies, with the majority published in the past 5 years. The effects of music interventions were categorized into 4 groups of health outcomes: positive (58 results), potentially positive (4 results), inconclusive (2 results), and no effect (3 results). The health outcomes were further classified into 5 groups: psychological well-being, cognitive functioning, physiological responses, quality of life, and overall well-being.

**Conclusions:**

The study revealed that music interventions for older adults can have positive or potentially positive effects on health outcomes, encompassing psychological well-being, cognitive functioning, physiological responses, quality of life, and overall well-being. However, some studies yielded inconclusive or no effect. The study offers valuable insights for healthcare professionals and serves as a visual resource to access evidence-based information on the use of music interventions in promoting health and addressing various conditions in older adults.

## 1. Introduction

The world’s population is aging rapidly, with older adults accounting for a significant proportion of the population. In China, the number of older adults has reached 264 million, comprising 18.7% of the total population.^[1]^ As the population ages, chronic diseases are becoming more prevalent, which places an increasing burden on families. Chronic diseases not only affect the physical health of older adults but also their psychological health, resulting in negative emotions such as anxiety, depression, and stress that significantly affect their quality of life.^[2]^

As the world’s population ages, the number of older adults living with chronic diseases such as Alzheimer’s Disease continues to rise. Alzheimer’s Disease is one of the most common progressive central nervous system degenerative diseases associated with aging, affecting over 50 million people worldwide.^[3]^ Furthermore, estimates suggest that the number of people living with dementia is expected to double every 20 years, reaching 131.5 million by 2050 and imposing significant personal, social, and economic burdens.^[4,5]^

Cognitive impairment poses significant challenges for individuals with dementia, leading to a decline in physical functioning, difficulties in rehabilitation, reduced independence in daily activities, decreased ability to cooperate with care, heightened agitation, and social isolation. These challenges place substantial burdens on caregivers and contribute to the prevalence of depression, isolation, and apathy among dementia patients. Consequently, families may opt for institutional care, placing additional strain on social healthcare resources and financial resources.

Non-pharmacological interventions have been shown to be safe and effective in improving the physical, emotional, psychological, social, and cognitive needs of older adults, thus improving their quality of life.^[6,7]^ Music therapy and music interventions are closely related concepts within the field of using music for therapeutic purposes. Music therapy is a specialized form of treatment that involves the professional practice of trained music therapists. It utilizes music and its elements to achieve specific therapeutic goals, such as enhancing communication, emotional expression, and cognitive functioning. Music therapists assess individuals’ needs and tailor interventions to address their unique circumstances. On the other hand, music interventions encompass a broader range of applications that utilize music and its elements for improving health and well-being. While music therapy is a specific discipline conducted by trained professionals, music interventions can be implemented by nonprofessionals, healthcare providers, volunteers, or other relevant professionals. Music interventions can include music therapy but also encompass other forms of music-based interventions such as music activities, music appreciation, and music training. Therefore, music therapy is a subset of music interventions, with the former involving specialized training and a therapeutic focus, while the latter encompasses a broader scope of music-related interventions.^[8]^ Music intervention has been shown to have beneficial effects on the cognitive, physiological (such as heart rate, blood pressure, respiratory rate, cortisol levels, immune function markers, and other relevant biomarkers), and psychological problems of older adults.^[9]^

In fact, research has shown that listening to and playing music can change brain functions, improving cognitive functions such as memory^[10]^ and attention,^[11]^ as well as behavioral symptoms of older adults.^[12]^ Long-term music training and learning of related skills can even stimulate the brain development of older adults.^[13]^ Music intervention has also been shown to alleviate negative emotions such as anxiety^[14]^ and depression,^[15]^ and activate the subcortical circuit, limbic system, and emotional reward system, thereby stimulating well-being and improving the quality of life.^[16]^ On the other hand, some studies report that the short-term effects of music intervention are limited in improving the cognitive function and emotion of older adults.^[17,18]^

Despite the existing reviews on the health benefits of music interventions for older adults, there is a need for a comprehensive synthesis and systematic evaluation of the available scientific evidence. The Global Evidence Mapping initiative, established in 2007, aims to identify knowledge gaps and future research needs through systematic and wide-ranging searches, presenting the results in a user-friendly format, such as visual graphs or searchable databases.^[19]^ This study aims to address this need by providing a clear and concise map of music prescriptions and research findings related to health outcomes for older adults. For this study, the Global Evidence Mapping initiative was utilized to conduct an Evidence Map on the impact of music interventions on the health of older adults. By conducting a rigorous review of the literature using standardized methodologies, we aim to overcome the limitations of previous studies and provide a more robust understanding of the effects of music interventions on the health of older adults. Our research seeks to bridge the gaps in the current knowledge by exploring the specific impacts of music interventions on cognitive functioning, psychological well-being, and overall health in older adults. The findings of this study will contribute to the existing body of knowledge, inform evidence-based practices, and potentially guide the development of clinical guidelines and future research studies in this field.

## 2. Method

The search strategy included systematic reviews, meta-analysis. The systematic reviews were conducted following the Preferred Reporting Items for Systematic Reviews and Meta-Analyses guidelines^[20]^ and the Evidence Map methodology^[21]^ to ensure a reliable summary of the best available evidence. Tableau was used to graphically display the number of reviews, intervention effects, confidence levels, and health outcomes.

### 2.1. Data sources

We conducted a comprehensive search for relevant studies across multiple databases, including PubMed, Web of Science, Embase, Cochrane, SinoMed, National Knowledge Infrastructure, and WanFang datebase, without any language restrictions, from the inception of each database to November 2022. The search included the use of keywords such as “systematic review” or “meta-analysis,” as well as “music intervention” or “therapy, music,” or “music* intervention*,” or “music,” in conjunction with terms such as “aged,” “older,” “older people,” “elderly,” or “older adults.” A combination of subject words and free words was used in the search strategy, and detailed search strategies can be found in Table 1.

**Table 1:** The retrieval strategy of this study.

Databases	No	Retrieve content	Number of retrieved results
PubMed	1	“aged” (MeSH Terms) OR (older[Title/Abstract]) OR (older people[Title/Abstract]) OR (elderly[Title/Abstract]) OR (older adults[Title/Abstract])	751,475
	2	“Music Therapy”[Mesh] OR (”Therapy, Music”[Title/Abstract]) OR (”music* intervention*”[Title/Abstract]) OR (Music[Title/Abstract])	21,277
	3	(“systematic review” [Title/Abstract]) OR (”meta-analysis”[Title/Abstract])	3354,184
	4	((“aged” (MeSH Terms) OR (older[Title/Abstract])OR (older people[Title/Abstract])OR (elderly[Title/Abstract])OR (older adults[Title/Abstract])) AND (“Music Therapy”[Mesh] OR (”Therapy, Music”[Title/Abstract]) OR (”music* intervention*”[Title/Abstract]) OR (Music[Title/Abstract]))) AND ((”systematic review” [Title/Abstract]) OR (”meta-analysis”[Title/Abstract]))	83
WOS	1	TS= (aged OR older OR older people OR elderly OR older adults)	2727,585
	2	TS= (Music Therapy OR Therapy, Music OR music* intervention* OR Music)	22,027
	3	TS= (systematic review OR meta-analysis)	397,705
	4	#1 AND #2 AND #3	206
Embase	1	*aged/exp	43,072
	2	aged.ab,ti	929,865
	3	older.ab,ti	726,599
	4	older people.ab,ti	42,639
	5	elderly.ab,ti	390,499
	6	older adults.ab,ti	129,715
	7	1 or 2 or 3 or 4 or 5 or 6	1765,651
	8	music therapy/exp	5029
	9	Therapy, Music.ab,ti	179
	10	“music* intervention*.”ab,ti	859
	11	Music.ab,ti.	24,290
	12	Music Therapy.ab,ti	4145
	13	8 or 9 or 10 or 11 or 12	24,874
	14	systematic review.ab,ti	292,188
	15	meta-analysis.ab,ti	273,563
	16	14 or 15	436,082
	17	7 and 13 and 16	107
Cochrane	1	aged	569,648
	2	(older):ab,ti,kw OR (older people):ab,ti,kw OR (elderly):ab,ti,kw OR (older adults):ab,ti,kw	112,034
	3	#1 or #2	617,727
	4	Music Therapy	3702
	5	(Therapy, Music):ab,ti,kw OR (music* intervention*):ab,ti,kw OR (Music):ab,ti,kw	6173
	6	#4 or #5	6442
	7	(systematic review):ab,ti,kw OR (meta-analysis):ab,ti,kw	24,465
	8	#3 and #6 and #7	111(reviews are 83)
Sinomed	1	“音乐疗法”[不加权:扩展]	7709
	2	“音乐干预”[常用字段:智能] OR “音乐”[常用字段:智能]	44,317
	3	(“音乐干预”[常用字段:智能] OR “音乐”[常用字段:智能]) OR (“音乐疗法”[不加权:扩展])	44,317
	4	“老年人”[不加权:扩展]	48,091
	5	“老年”[常用字段:智能] OR “老人”[常用字段:智能]	1246,308
	6	(“老年”[常用字段:智能] OR “老人”[常用字段:智能]) OR (“老年人”[不加权:扩展])	1246,308
	7	“系统评价”[常用字段:智能] OR “meta分析”[常用字段:智能] OR “荟萃分析”[常用字段:智能]	254,384
	8	(“系统评价”[常用字段:智能] OR “meta分析”[常用字段:智能] OR “荟萃分析”[常用字段:智能]) AND ((“老年”[常用字段:智能] OR “老人”[常用字段:智能]) OR (“老年人”[不加权:扩展])) AND ((“音乐干预”[常用字段:智能] OR “音乐”[常用字段:智能]) OR (“音乐疗法”[不加权:扩展]))	4
CNKI	1	(主题=音乐疗法) OR (主题=音乐干预) OR (主题=音乐)	660,199
	2	((((主题%=“音乐疗法”or 题名%=”音乐疗法”)OR(主题%=”音乐干预”or 题名%=”音乐干预”))OR(主题%=”音乐or 题名%=”音乐”))AND(((主 题%=”老年人”or 题名%=”老年人”)OR(主题%=老年”or 题名%=”老年”))OR(主题%=”老人”or 题名%=”老人”)))	2074
	3	(((((主题%=“音乐疗法”or 题名%=”音乐疗法”)OR(主题%=”音乐干预”or 题名%=”音乐干预”))OR(主题%=”音乐”or 题名%=”音乐”))AND(((主 题%=”老年人”or 题名%=”老年人”)OR(主题%=”老年”or 题名%=”老年”))OR(主题%=”老人”or题名%=”老人”)))AND(((摘要=系统评价”)OR(摘要=”meta分析”))OR(摘要=”荟萃分析”)))	8
Wanfang	1	主题:(音乐疗法+音乐干预+音乐)	772,843
	2	主题:(老年人+老年+老人)	1068,071
	3	主题:(系统评价+荟萃分析+meta分析)	616,381
	4	主题:(音乐疗法+音乐干预+音乐) and 主题:(老年人+老年+老人) and 主题:(系统评价+荟萃分析+meta分析)	22

### 2.2. Inclusion criteria

#### 2.2.1. Design:

Systematic reviews focused on music intervention for older adults, which self-identified as a “systematic review” or “meta-analysis” that reported the search sources and accounted for identified studies, were eligible for inclusion.

#### 2.2.2. Population:

Systematic reviews of older adults aged 60 years and older, the majority of the participants were considered to be older adults according to the Chinese definition,^[22]^ regardless of their health status, were eligible for inclusion. Studies that did not focus on music intervention for older adults were excluded.

#### 2.2.3. Intervention:

Systematic reviews of the effects of music intervention for older adults, including combination therapies incorporating music intervention, were eligible for inclusion. Systematic reviews that did not systematically search for music intervention studies and reviews were excluded.

#### 2.2.4. Comparison:

Comparison included pharmacological treatments, usual treatments, and placebo treatment.

#### 2.2.5. Outcomes:

Systematic reviews reporting on health outcomes of older adults were eligible for inclusion. Specifically, we focused on the effects of music interventions on the psychology, cognition, physiology, quality of life, and well-being of older adults. Systematic reviews of acceptance, prevalence, costs, and unreported study design characteristics or patient health outcomes were excluded.

### 2.3. Procedures

**Figure 1.**
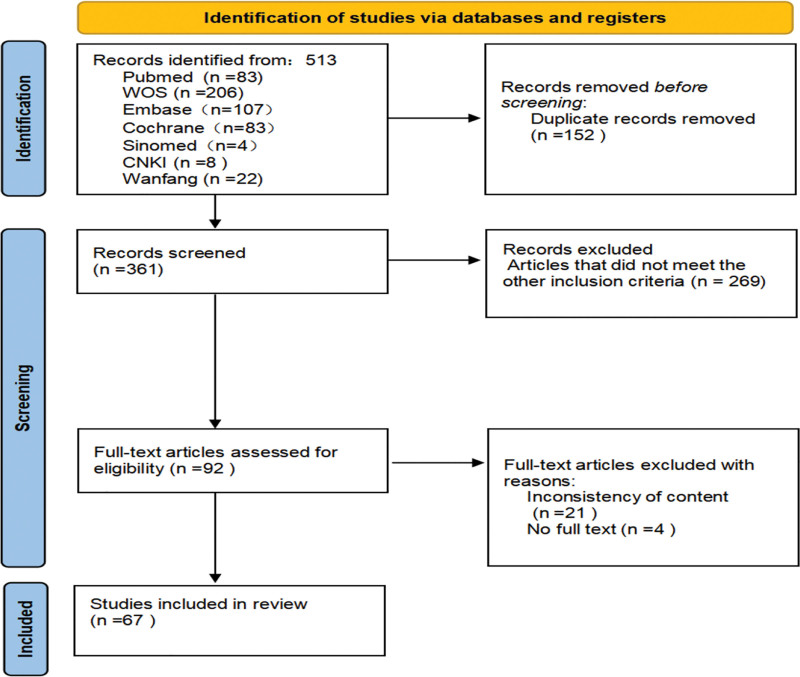
PRISMA 2020 flow diagram for new systematic reviews which included searches of databases and registers.

To select eligible studies, all identified hits were imported into Endnote (Version X9). Two independent reviewers, screened all the systematic reviews. The full-text publications were also screened by 2 independent reviewers according to the specified inclusion criteria. Firstly, duplicate records were removed manually and by software. Secondly, the titles and abstracts of the remaining records were examined to exclude irrelevant documents. Finally, the full texts of the remaining studies were retrieved for further screening. Disagreements were resolved by consensus, and if necessary, an additional reviewer was consulted. The reasons for exclusion of full-text publications were recorded and presented in Figure 1.

### 2.4. Methodological quality assessment

A MeaSurement Tool to Assess systematic Reviews (AMSTAR 2 tool)^[23]^ was utilized to assess the methodological quality of the systematic reviews included in this study. This tool is composed of 16 items, with 7 critical items (items 2, 4, 7, 9, 11, 13, and 15). For each of these items, the responses “Yes” (Y), “Partial Yes” (PY), or “No” (N) were used to evaluate specific questions. The overall confidence in each item was then classified as “Critically Low” (CL, “more than one critical flaw with or without non-critical weaknesses”), “Low” (L, “one critical flaw with or without non-critical weaknesses”), “Moderate” (M, “more than one non-critical weakness”), or “High” (H, “No or one non-critical weakness”).

### 2.5. Data extraction

Data extraction was performed by 2 independent reviewers who read all articles and extracted baseline information according to predefined criteria. This information included the article’s title, first author, year of publication, whether it mentioned Preferred Reporting Items for Systematic Reviews and Meta-Analyses or other reporting guidelines, number of randomized controlled trials, sample size, interventions, comparisons, outcomes, quality assessment tool of included primary studies, effects (summarized according to the views of the author of the original document), funding source, and AMSTAR2 rating overall confidence. Methodological quality assessments were also conducted. Disagreements between the reviewers were resolved through consensus, and a third reviewer was consulted when necessary.

## 3. Results

After the initial search yielded 513 citations, 67 studies ultimately met the inclusion criteria. In total, 209 unique outcomes were identified, with some studies reporting more than one relevant outcome. Furthermore, a single article may have included information on different populations, multiple types of music interventions, and more than 2 outcomes. Of the 67 included studies, there were 21 systematic reviews with meta-analysis, 33 systematic reviews without meta-analysis, and 13 meta-analysis. In this evidence map, we included 67 studies published in the past 2 decades that evaluated 106,253 older adults (only the number of subjects proposed by the author in the article is included). We observed a significant increase in the number of publications over time, with 45 of the 67 included studies published in the last 5 years. This suggests a growing interest in understanding how music impacts the health of older adults.

### 3.1. Quality of the included systematic reviews

Regarding the quality assessments of the overall confidence level for each systematic review, most studies showed a low level of confidence (n = 23 studies), indicating limitations in the methodology or reporting of the systematic review. Fifteen articles each were classified as having a high or moderate confidence level, suggesting more rigorous methodology and reporting. Fourteen studies were rated as having a critically low level of confidence, indicating serious flaws in the methodology or reporting.

### 3.2. Population

The majority of the systematic reviews included in this study focused on older adults or individuals diagnosed with cognitive impairment or dementia (n = 49 studies), while 11 systematic reviews included patients with mental disorders, and 7 systematic reviews included other types of older adults such as those with sleep disorders or healthy older adults. Table 2 provides an overview of the main characteristics of the sixty-seven systematic reviews, including sample size, patient characteristics, interventions, and primary outcomes.

**Table 2:** The main characteristics of the sixty-seven systematic reviews.

No	First author	Country	Year of publication	Mention of prisma	Populations	Number of RCTs	Sample size	Interventions	Comparison	Outcomes	Quality assessment tool	Effect	Fund	Amstar2 rating overall confidence
1	Peter Hoang	Canada	2022	Y	Adults aged 65 years or older	70	8259	Animal therapy, psychotherapy or cognitive behavioral therapy, multi component, counseling, exercise, music therapy, occupational therapy, reminiscence therapy, social interventions, and technological interventions	Individual animal therapy	Loneliness	BMJ best practice grading of recommendations assessment, development and evaluation of evidence tool	No effect	N	H
2	Kayla Atchison	Canada	2022	Y	Older adults living in long-term care (LTC)	80	NA	Music	Usual care, social interaction	Anxiety symptoms	Cochrane rob 2 tool	Positive effect	N	M
3	Claire V. Burley	Australia	2022	Y	Dementia	37	2636	Education training, therapeutic activities, cognitive rehabilitation or cognitive stimulation, reminiscence-based, physical activity, music, and other approaches	Pharmacological intervention	Depression	Several different previously used tools	Potentially positive	N	H
4	Nigussie Tadesse Sharew	Ethiopia	2022	Y	Older people with dementia	19	NA	Physical exercise, music, and cognitive interventions	Unimodal non-pharmacological interventions or control group with no intervention	Cognitive function	Robins-i tool for non-randomized control trial studies and the Cochrane risk-of-bias tool for randomized trials (rob 2)	Positive effect	N	H
5	Bai Zhifan	China	2022	N	The elderly in pension institutions	16	1039	Pet therapy, comprehensive psychological intervention therapy, cognitive behavior therapy, music therapy, reminiscence therapy and problem solving therapy	General health education	Depressive symptoms	Cochrane handbook	Positive effect	N	L
6	Catherine Jordan	Ireland	2022	Y	Age range 60–85 years with mild cognitive impairment	9	586	Music interventions	Usual care	Cognitive function and/or behavior, cognitive domains included executive function, visuospatial function, working memory, attention, verbal fluency and memory, behavioral domains included measures of depression, apathy, anxiety, and quality of life	The evidence project risk of bias tool	Potentially positive	Y	M
7	Teerapon Dhippayom	Thailand	2022	Y	Older adults aged ≥ 60 years	15	1144	Active music therapy, receptive music therapy, music medicine	Usual care	Depression	Grade assessment	Positive effect	N	M
8	Hui-Fen Hsu	China	2022	Y	Older adults aged 65 and older	8	524	Live music, recorded music, and active music, with a variety of music styles and genres	Usual care	Chronic pain	JBI	Potentially positive	Y	M
9	Erika Ito (mean age ranged from 60 to 87 years old	Japan	2022	Y	Men and women aged 60 + with a clinical diagnosis of cognitive im pairment or dementia	19	1024	Music-based intervention or community music activity including listening to music, singing, playing an instrument, and music with movement or exercise	No intervention/usual care, meditation, pharmacological intervention, exercise intervention, late intervention, and painting or other art related activities	General cognitive function, the frontal assessment battery (executive function), and the auditory verbal learning test (episodic memory)	Consolidated standards of reporting trials (consort) statement	Positive effect	Y	L
10	Zhao Yiran	China	2021	N	Age > 60 years old; simple mental state examination (MMSE) > 24)	10	397	Music intervention	Usual care	Cognitive function, executive function, memory and attention	JBI	Positive effect	Y	M
11	Zhi Hui Fong	China	2021	Y	Older persons aged 60 with mci	11	817	Arts-based, which includes dance/movement, drama, music, or visual arts	Appropriate control group (e.g., age matched, mci status, no treatment/waitlist/active control)	Global cognition, learning and memory, complex attention, executive functioning, language, and perceptual-motor function	Cochrane rob 2 tool	Inconclusive effect	Y	L
12	Ya-Jing Chen	China	2021	Y	Participants aged 65 years and older with a primary diagnosis of any depressive disorder	35	3797	Intervention classes (psychosocial, psychotherapy, physical activity, combined, treatment as usual) and individual intervention	Treatment as usual (usual care, no intervention, waiting list treatment), or active non-pharmacological intervention	Depression	Cochrane handbook	Positive effect	Y	L
13	Yo-Jen Liao Bsn	USA	2021	Y	Pain in people living with dementia	11	486	Massage, ear acupressure, music therapy, painting and singing, personal assistive robot, exercise, social activities, cognitive behavioral therapy, reflexology, tailored pain intervention, play activity, and person-centered environment program	Routine pain mausual caregement	Pain	The johns hopkins nursing evidence-based practice research evidence appraisal tool	Positive effect	N	H
14	Ma mengning	China	2021	N	Elderly patients with dementia	15	1101	Passive music intervention, passive music intervention, group intervention	Treatment as usual	Cognitive function	Cochrane handbook	Positive effect	Y	L
15	Sekyung Jang	Ireland	2021	N	Older adults (age: 60 or older)	20	NA	Types of music experiences reported in the selected studies were singing, movement to music, music listening, instrument play, improvisation, music-guided reminiscence, song writing, music guided relaxation, guided imagery to music, and instrument making	Usual care	Depressive symptoms, general mood states, stress and relaxation, affective disturbance, decrease in negative mood, increase in positive mood, expression of positive emotion, dealing with difficult emotions such as frustration and sadness, and self-confidence and shared feelings of joy	Standardized assessment forms (clear-npt)	Positive effect	N	M
16	Chia-Te Chen	China	2021	Y	Older adults aged 60 years and older	5	288	Music intervention	Standard care (or no treatment)	Sleep quality	Cochrane handbook	Positive effect	N	L
17	Cong Wang	China	2021	Y	Older adults	9	489	Music interventions, including passive and active music interventions	No control group, blank and waitlist control group, usual care or other interventions	Sleep quality, sleep latency, sleep duration, sleep efficiency and sleep of daytime dysfunction	Cochrane risk of bias (rob)tool for RCTs and the risk of bias in non-randomized studies - of interventions (robins-i) tool for non-RCTs	Positive effect	Y	L
18	Jennie L. Dorris	USA	2021	N	Older adults with probable mci and mild or moderate dementia	21	1742	Cognitive functioning, emotional well-being, and social engagement	Exercise	Cognitive functioning	Cochrane rob 2 tool	Positive effect	Y	L
19	Wang Ni	China	2021	N	Senile dementia patients	15	830	Music intervention	Conventiousual carel treatment	Cognitive function, depressive state and self-care ability	Cochrane handbook	Positive effect	Y	L
20	Ying-Quan Wang	China	2020	N	People with mild cognitive impairment (MCI)	25	2245	Physical exercise, cognitive stimulation, cognitive training, cognitive rehabilitation, musical therapy and multi-domain interventions	Pharmacotherapy	Cognition	Cochrane handbook	Positive effect	N	H
21	Claudia Meyer	Australia	2020	Y	Dementia	38	NA	Cognitive stimulation, environment, exercise, mealtimes, montessori, music, psychological treatment, reminiscence therapy, sensory stimulation, aromatherapy, light therapy, therapeutic touch, simulated presence therapy, transcutaneous electrical nerve stimulation	Usual care	Responsive behaviors, maintenance/improvement in functional capacity and/or co-morbid emotional disorders	Amstar tool	Potentially positive	N	M
22	Shouchao Wei	China	2020	N	The global cognition dysfunction associated with Alzheimer’s disease	25	3238	Physical activity, transcranial magnetic stimulation therapy, cognition-based therapy, reminiscence therapy, acupuncture therapy, music therapy, food therapy	Usual care	Cognitive function	Grade	Positive effect	Y	H
23	Minah Amor Gaviola	Australia	2020	Y	Dementia	4	NA	Individualized music listening	Other music and non–music-based interventions	Agitation, anxiety and depression and physiological outcomes, cognitive function and quality of life	Cochrane’s collaboration tool	Positive effect	N	M
24	Celia Moreno-Morales	Spain	2020	Y	People living with dementia	8	816	Music	Usual care	Cognitive function, quality of life, and/or depressive state	Physiotherapy evidence database (pedro) and critical appraisal skills program (casp) scales	Positive effect	N	L
25	Lídia Sousa	Portugal	2020	Y	Patients with dementia	9	246	Music-based intervention	Treatment as usual	Well-being, mood, engagement/relationship and global cognitive function, as well as a reduction in behavioral and psychological symptoms of dementia, resistive care	The downs and black checklist	Positive effect	N	CL
26	Jennifer A. Watt	Canada	2021	Y	People with dementia	213	25177	Non-drug interventions	Usual care or any other intervention	Depression	Cochrane risk of bias tool	Positive effect	Y	H
27	Deirdre Noone	UK	2019	N	People with dementia	8	NA	Psychosocial interventions that specifically targeted depression or anxiety symptoms	Treatment as usual	Depression or anxiety	Cochrane’s risk of bias tool	Positive effect	N	M
28	Christine Brown Wilson	UK	2019	N	Older people with dementia Parkinson’s disease with dementia	13	NA	Music therapy and activity-based interventions	Usual mausual caregement	Depression or anxiety	Cochrane’s risk of bias tool, consort 2010 guidelines	Positive effect	Y	M
29	Higuti, A. Y.	Brazil	2019	N	Alzheimer’s patients	10	NA	Music-related physical exercise (with or without)	Usual care	Physical exercise, cooperation and interaction	Pela escala de avaliação pedro	Positive effect	N	H
30	Deirdre Fetherstonhaugh	Australia	2019	Y	People aged over 65 years with dementia	20	NA	Animal therapy and music therapy	Routine care	Mealtime function	JBI	Positive effect	Y	M
31	Liang Jinghong	China	2019	N	People with mild to moderate Alzheimer’s disease (ad)	19	3768	Pharmacotherapy, cognitive stimulation, cognitive rehabilitation, computer cognitive training, music treatment, physical exercise, nursing intervention	Placebo	Cognitive ability	Cochrane handbook	Positive effect	N	CL
32	Jinghong Liang	China	2019	N	Elderly adults with dementia	68	9937	Physical exercise, computerized cognitive training, music therapy, cognitive stimulation therapy, cognitive rehabilitation, nursing therapy, psychosocial therapy, pharmacological therapy and control group	Treatment as usual, placebo	Cognitive ability	Cochrane handbook	Positive effect	N	M
33	Soo Ji Kim	South Korea	2019	N	Older adults, ages 60 years and older, and any clinical diagnosis of cognitive impairment had to be due to aging	10	635	Music intervention	Usual care	Memory, working memory, recall, executive function, visuospatial perception, verbal fluency, attention, attentional control, and processing speed	Cochrane handbook	Positive effect	Y	L
34	Hui-Chi Li	China	2019	N	People with dementia	7	NA	Music intervention	Non-music therapy intervention	Depression	Jadad scale	Positive effect	N	CL
35	Han Qiao	China	2019	N	Dementia patients, >60	8	462	Music intervention	Usual care	Agitation	Cochrane	Positive effect	Y	L
36	Kelvin K. F. Tsoi	China	2018	Y	People with dementia	38	1418	Music intervention	Usual care included usual or standard care	Cognitive function, apathy, anxiety, depressive symptoms, agitation, and other behavioral problems	Cochrane’s risk of bias	Positive effect	N	M
37	Jing-Hong Liang, Bsc	China	2018	Y	Older adults with Alzheimer disease or mild cognitive impairment	20	1931	Physical exercise, music therapy, computerized cognitive training (non-pharmacological therapies), and nutrition therapy (pharmacological therapy)	Control group alone or in any combiusual caretion	Cognition	Cochrane collaboration	No effect	N	L
38	Laura E. Legere	Canada	2018	Y	Dementia in older adults	18	NA	Music therapy, interventions targeting pain, person-centered approaches, and education for family caregivers	Usual care	Behavioral and psychological symptoms	Amstar	Potentially positive	Y	L
39	Richard Olley	Australia	2018	Y	Patients with dementia	85	NA	Non-pharmacological therapy	Usual care	Agitation characteristics, quality of life and behavioral symptoms, progression of functional disability, depression, psychosocial	Standard quality assessment score (sqas)	Positive effect	N	CL
40	Amy Curtis	UK	2018	Y	Older adults	71	2086	Arts-based intervention	Another activity	Physical health, cognition, quality of life, psychological wellbeing, behavioral changes and agitation	Grade	Potentially positive	N	L
41	S. Ronzi	UK	2018	Y	Older adults aged 60 + years	40	NA	Mentoring, intergenerational and multi-activity programmes, dancing, music and singing, art and culture and information-communication technology	Usual care	Quality of life and measures of wellbeing, cognitive function, autonomy and physical activity	Liverpool quality assessment tools	Positive effect	Y	H
42	Jenny T. van der Steen	Netherlands	2018	N	People with dementia	22	1097	Music-based therapeutic interventions	Usual care or other activities with or without music	Depressive symptoms, behavioral problems, emotional wellbeing, quality of life and anxiety	Grade	Positive effect	N	H
43	Hanneke van der Wal-Huisman	Netherlands	2018	Y	Hospitalized surgical postoperative patients (no outclinic surgery)); age (mean) ≥ 60 years;	17	NA	Investigating music	Usual care	Pain and anxiety, relaxation, cognitive functioning and satisfaction	NA	Positive effect	N	CL
44	Laura Fusar-Poli	Italy	2018	Y	Patients with dementia	6	110	Music therapy	Standard care, or other nonmusical types of intervention	Global cognition, complex attention, executive function, learning and memory, language, and perceptual-motor skills	Cochrane’s tool	Positive effect	N	H
45	Lauren Istvandity	Australia	2017	N	Older people	19	NA	Music therapy and reminiscence therapy	Usual care	Cortisol levels and blood pressure, stress, anxiety, and depression, life satisfaction	NA	Positive effect	N	CL
46	M. Gómez-Romero	Spain	2017	N	Patients with dementia	11	NA	Music therapy	Usual care	Behavior disorders, anxiety and agitation	NA	Positive effect	N	CL
47	Yingshi Zhang	China	2017	Y	Older adults with dementia	34	1757	Music therapy	No music care	Disruptive behavior and cognitive function, the secondary outcomes included depressive score, anxiety score and quality of life	Pedro and casp scale scores, physiotherapy evidence database (pedro) scale score	Positive effect	N	H
48	Bing Xu	China	2017	Y	Older adults	10	966	Music therapy	Without music	Cognitive function, disruptive behavior, depressive score, anxiety score, quality of life	Pedro scale score and casp scale score	Positive effect	N	H
49	Catherine Travers	Australia	2016	N	People living with dementia	34	NA	Recreational activities, reminiscence therapy, music therapy interventions, training staff to develop individual care plans using person-centered care or similar approaches, animal-assisted therapy, multi-sensory interventions and social interaction	Usual care	Quality of life, agitation, aggression, depression, wandering and apathy, mood, function, cognition and sleep	Grade	Positive effect	Y	M
50	Vicky Booth	UK	2016	N	Older persons who were 65 years or older	8	1041	Physical and cognitive activities, music-based group exercise and mind-body tai chi	Usual care	Falls, including falls rate, specific falls risk measures or related clinical outcome measures	Joanna briggs institute meta-analysis of statistics assessment and review instrument (JBI-mastari) software	Positive effect	N	L
51	K. Zhao	China	2016	N	Elderly	19	NA	Music therapy	No treatment, standard therapies, or an active control condition	Depression	Cochrane collaboration’s risk of bias tool	Positive effect	Y	L
52	Millan-Calenti, J. C.	Spain	2016	N	Alzheimer’s disease (AD) patients aged 65 years and above	8	NA	Non-pharmacological interventions	Usual care	Agitation	NA	Positive effect	N	CL
53	Alexandra Martini de Oliveira	Brazil	2015	N	Older adults	20	NA	Activities, music therapy, aromatherapy, exercises, light therapy, touch therapy, combination of activities, cognitive rehabilitation	Usual care	Behavioral and psychological symptoms of dementia	NA	Positive effect	Y	CL
54	Piyanee Klainin-Yobas	Singapore	2015	N	Older adults	15	NA	Progressive muscle relaxation training, music intervention, and yoga	Usual care	Anxiety and depression	Data collection form guided by Cochrane’s systematic review	Positive effect	Y	L
55	Avis R. Ing-Randolph	USA	2015	N	65 years and older	7	NA	Roup music interventions	Usual care	Anxiety	NA	Potentially positive	N	CL
56	Hui-chi li	China	2015	N	Older adults over the age of 60	12	234	Music therapy intervention	Usual care	Cognitive function	Consolidated standards of reporting trials (consort) statement	No effect	N	L
57	Yu-Shiun Chang	China	2015	Y	People with dementia	10	NA	Music therapy	Usual care	Disruptive behaviors, anxiety levels, depressive moods and cognitive functioning	Cochrane collaboration’s tool	Positive effect	Y	H
58	Rie Konno	Japan	2014	N	Older people with dementia	19	NA	Non-pharmacological intervention	Usual care	Disruptive behavior, problem behavior, agitation, aggression and resistance-to-care	JBI	Potentially positive	N	L
59	Gill Livingston	UK	2014	Y	Older adults with dementia	2	NA	Person-centered care, communication skills and dementia care mapping, sensory therapy activities, and structured music therapies	Usual care	Agitation	Consolidated health economic evaluation reporting standards (cheers) checklist	Positive effect	Y	H
60	Li, Y. H	China	2014	N	Elderly with dementia	18	NA	Music therapy	Usual care	Cognitive, mental symptoms and dietary problems	NA	Positive effect	N	CL
61	Tomomi Ueda	Japan	2013	N	Patients with dementia	23	NA	Music therapy	Usual care	Behavioral and psychological symptoms of dementia, cognitive function, and activities of daily living, depression, anxiety, and behavioral symptoms such as agitation, apathy, elation, and irritability	Grade	Positive effect	N	L
62	Ieva Vasionyt	Lithuania	2013	N	Patients with dementia	19	478	Music therapy	Usual care	Behavioral, cognitive and physiological outcome measures, and medium effects on affective measures	Effect sizes	Positive effect	N	L
63	Jiménez-Palomares, M.	Spain	2013	N	Patients over 65 years of age with moderate dementia	10	NA	Music therapy	Usual care	Behavioural and cognitive functioning and social participation	NA	Positive effect	N	CL
64	Imogen N. Clark	Australia	2012	Y	Older adults	12	309	Activity programs, music, behavior therapy, light therapy, carer education and changes to the physical environment	No-music interventions	Physical activity	Pedro scale	Positive effect	N	L
65	Janet Opie	Australia	1999	N	People with dementia	43	NA	Music interventions	Usual care	Behaviour disorders	NA	Positive effect	N	CL
66	Darina Petrovsky	USA	2015	N	Older adults with mild dementia	10	NA	Music interventions	Usual care	Anxiety and depression	NA	Inconclusive effect	N	CL
67	Jennifer A. Watt	Canada	2019	N	Adults with dementia	163	23143	Multidisciplinary care, massage and touch therapy, music combined with massage and touch therapy, recreation therapy	Usual care	Reducing aggression and agitation	Cochrane’s risk of bias tool	Positive effect	Y	M

AMSTAR2 Rating overall confidence: CL= “Critically Low,” L =“Low,” M=“Moderate,” H=“High”.

### 3.3. Years of publication and country

**Figure 2.**
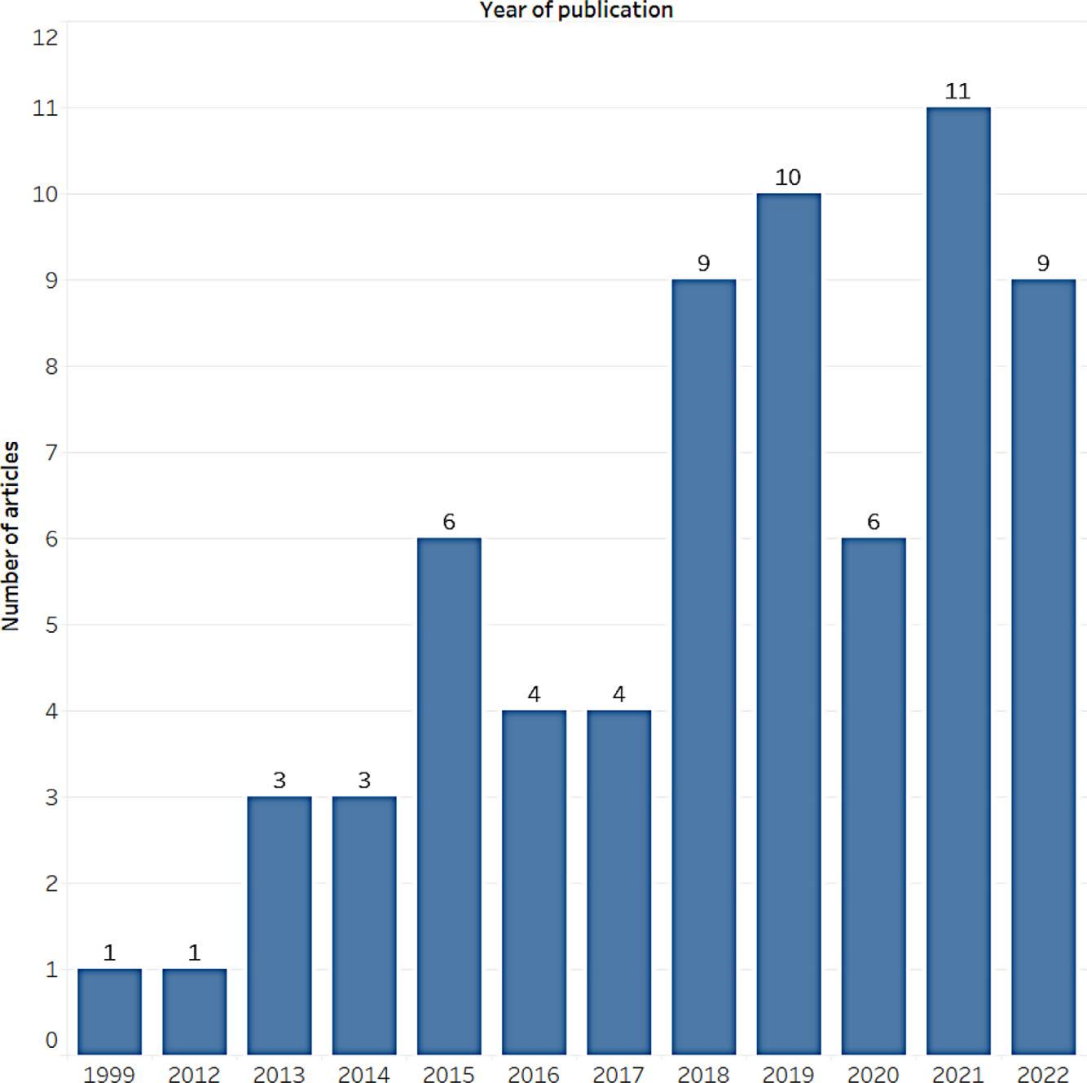
Annual publication volume of articles.

**Figure 3.**
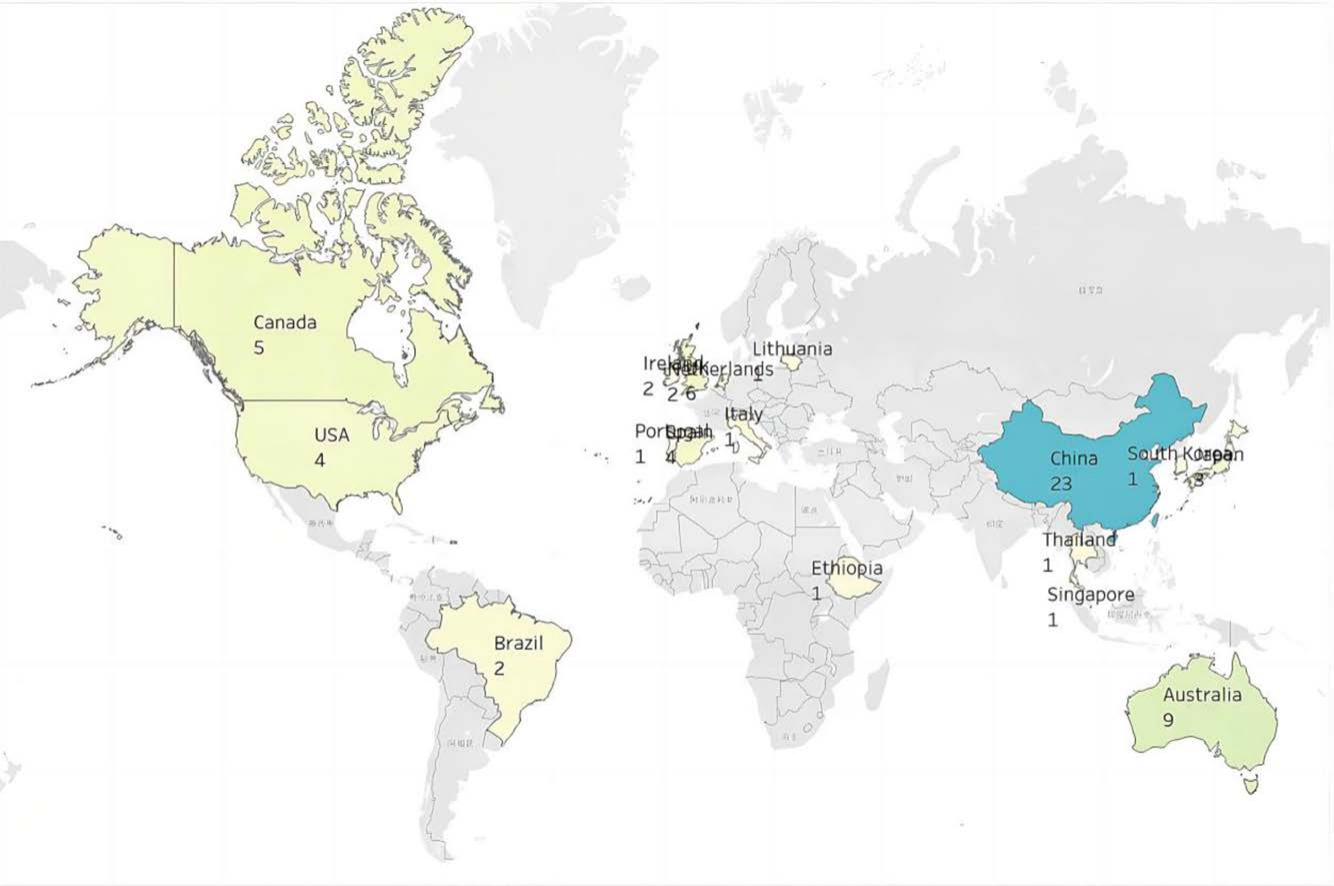
Distribution of articles by country.

The majority of the studies (n = 45) were published between 2018 and 2022 (Fig. 2). With respect to the regions and countries, 29 studies were conducted in Asia, 17 in Europe, 9 in North America, 9 in Oceania, 2 in South America, and 1 in Africa. China had the highest number of articles published in this study, with 23 articles, followed by Australia (n = 9), the UK (n = 6), and Canada (n = 5) (Fig. 3).

### 3.4. Outcomes and effects

**Figure 4.**
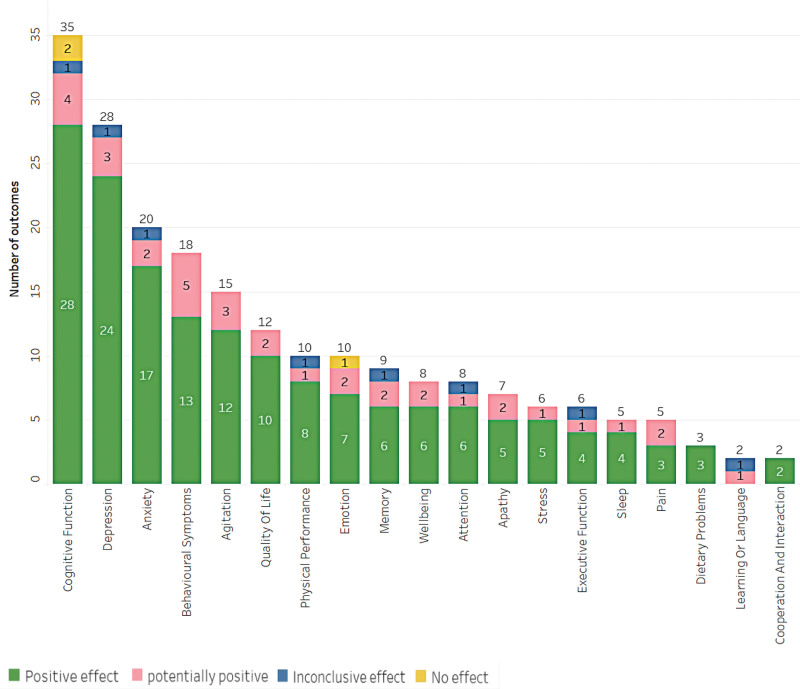
Main outcomes categorized by results and confidence level.

**Figure 5.**
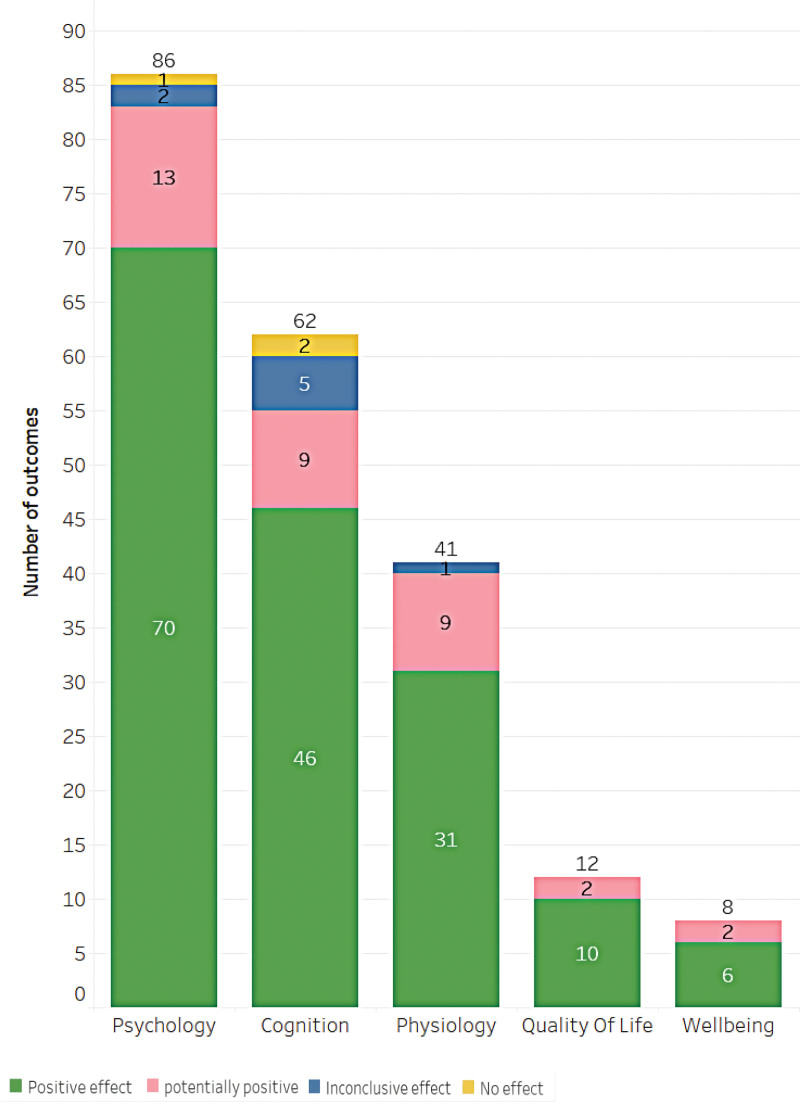
Results of music effects by category in the research included in the Evidence Map.

**Figure 6.**
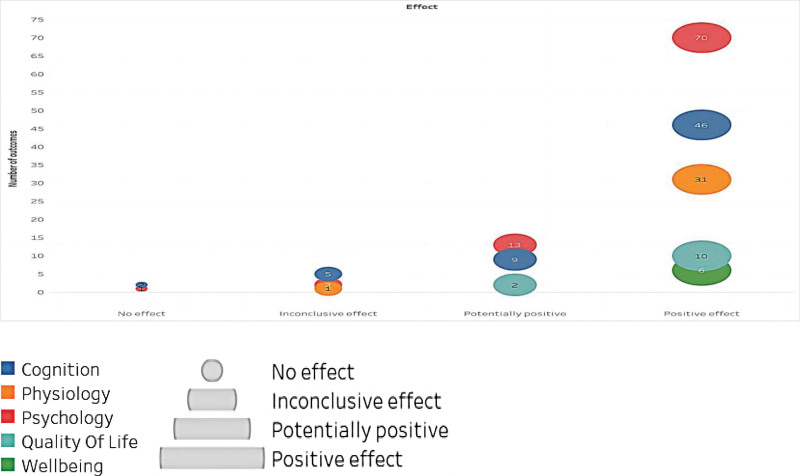
The effect of each result.

This study examined the use of music as an intervention for various health conditions. Within the 67 studies included, effects were classified into 4 categories of health outcomes: positive (58 results), potentially positive (4 results), inconclusive (2 results), and no effect (3 results), as shown in Figure 4. It should be noted that the confidence levels reported in this study refer to the quality of the included systematic reviews, not the efficacy of the music interventions. Figure 5 depicts the results, which are divided into 5 main categories: psychology, cognition, physiology, quality of life, and well-being. The effect of each result is shown in Figure 6, with the size of each bubble corresponding to the magnitude of the effect size. However, it is important to note that the direction of the effect (i.e., positive or negative) is not provided in this figure. A more detailed discussion of the outcomes and effects is provided in the following section.

### 3.5. Psychology

**Figure 7.**
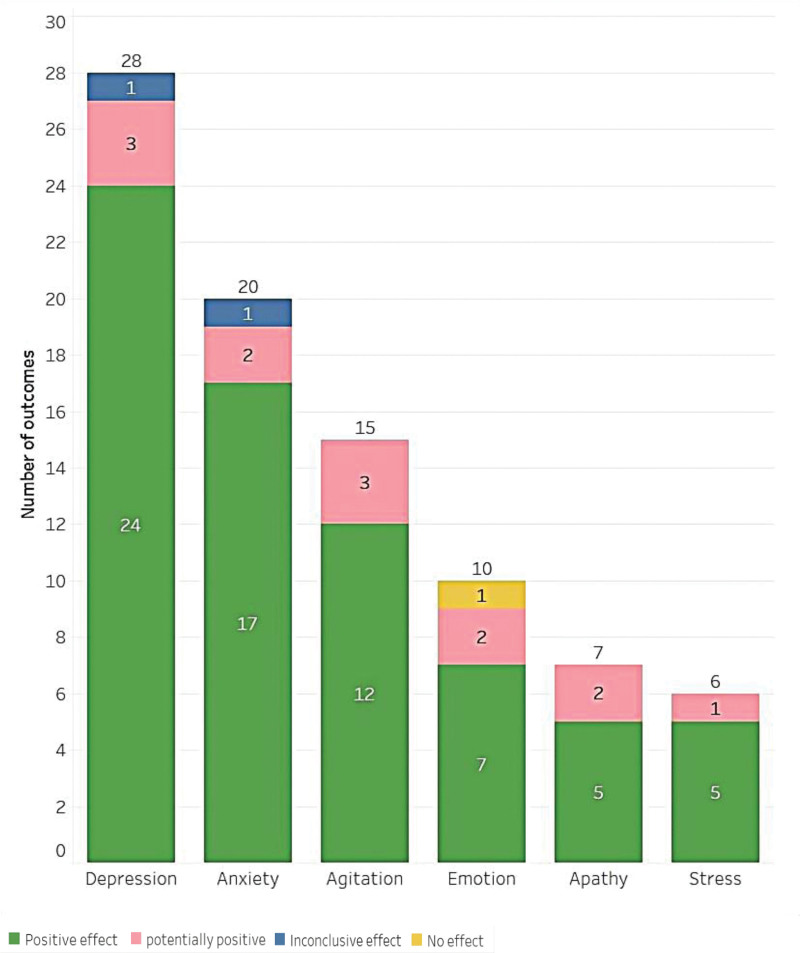
Main outcomes in the category of psychology.

The psychology category consisted of 86 studies that examined the impact of music practices. Among these studies, 70 reported positive effects, 13 reported potentially positive effects, 2 reported inconclusive effects, and 1 did not provide information on the effects. The majority of the studies focused on the treatment of depression, anxiety, agitation, emotion, and apathy, with stress being the next most commonly investigated area (Fig. 7). The top 3 areas with the highest number of studies were depression (28 studies), anxiety (20 studies), and agitation (15 studies). In terms of the population, 9 studies included patients with a mental disorder diagnosis, and 83 studies examined the impact of music practices on older adults.

### 3.6. Cognition

**Figure 8.**
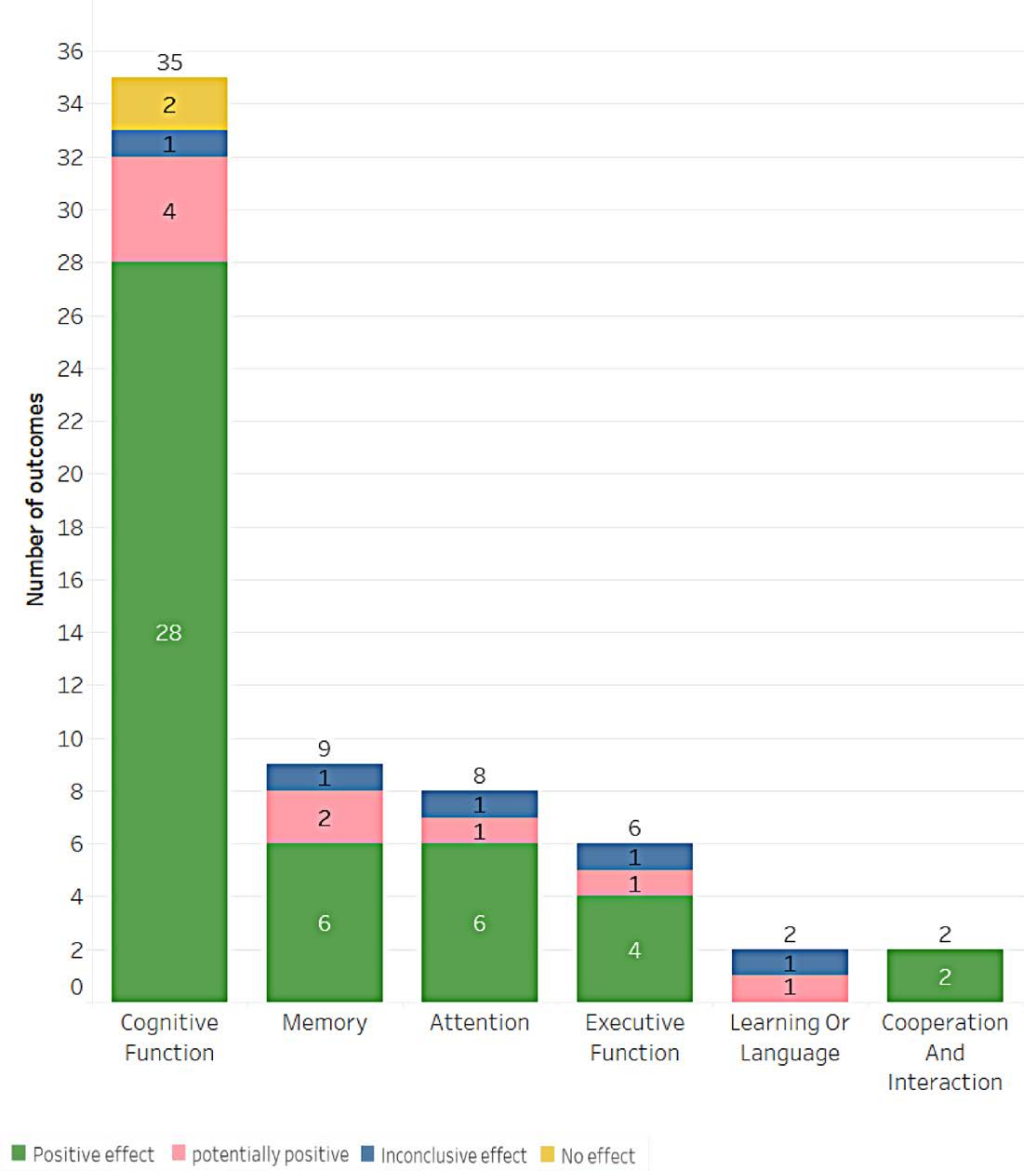
Main outcomes in the cognition category.

Regarding cognitive function outcomes, the results of this study show a total of 62 outcomes. Of these, 46 showed a positive effect of music intervention, 9 had a potentially positive effect, 5 were inconclusive, and 2 showed no effect. The most common outcomes were improvements in cognitive function, memory, attention, executive function, learning, language, cooperation, and interaction (as shown in Fig. 8). The top 3 outcomes were improvements in cognitive function (35 results), memory (9 results), and attention (8 results), respectively. It is important to note that these results only apply to studies that met the inclusion criteria and had intended outcomes. Additionally, discussing the outcomes of the studies would provide more valuable insights into the effectiveness of music interventions for cognition.

### 3.7. Physiology, well-being, and quality of life

**Figure 9.**
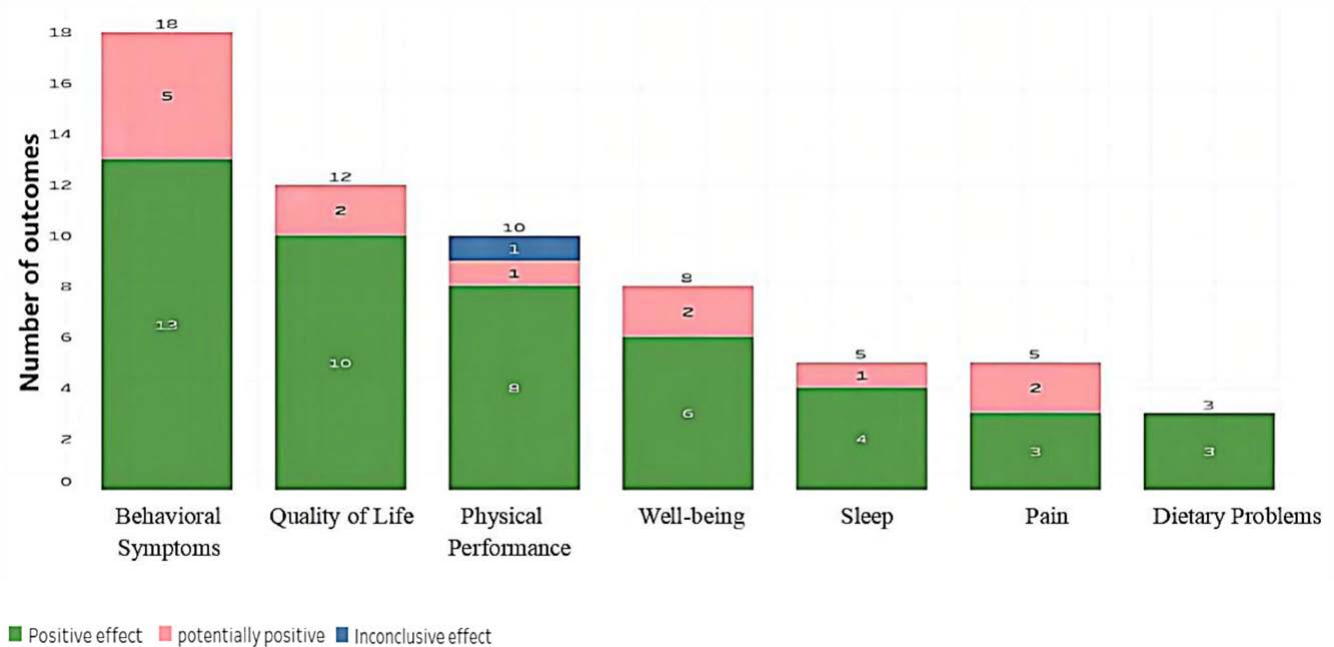
Main outcomes in the physiology, well-being, and quality of life category.

Out of the 61 results obtained for the practice of music (Fig. 9), 47 were classified as positive, 13 as potentially positive, and one as inconclusive. Positive outcomes refer to improvements in health or well-being, potentially positive outcomes indicate some evidence of improvement but with some uncertainty, and inconclusive outcomes suggest that there is not enough evidence to draw a conclusion. The most common areas of improvement were behavioral symptoms, quality of life, physical performance, well-being, sleep, pain, and dietary problems, with 18, 12, and 10 results, respectively. It is worth noting that positive outcomes indicate that music intervention had a beneficial effect on the outcome measures.

## 4. Discussion

The psychological health of older adults, especially anxiety and depression, has attracted the attention of many authors. The map shows that depression and anxiety were the disorders with the highest number of musical intervention outcomes, with 28 and 20 results, respectively. Many studies have shown that music intervention, compared with usual intervention, is not only conducive to improving the anxiety and depression of normal older adults, but also to improving them in older adults with dementia.^[15]^ Music intervention compared with social interaction^[13]^ and pharmacological intervention^[24]^ were found to improve anxiety and depression symptoms. Based on the evidence map, we suggest that music intervention can be used as one of the forms of integrative and complementary treatment for the psychological health of older adults.

Moreover, Dhippayom et al^[25]^ proposed that although active music intervention of more than 60 minutes a week is the most effective intervention to alleviate the depression of older adults, listening to music that older adults like for 60 minutes or more a week is an alternative. This study revealed that group-based music interventions were effective in reducing anxiety and depression in older adults, consistent with the findings of Ing-Randolph’s study.^[14]^ The duration of the intervention was found to be a crucial factor in determining its efficacy. Specifically, interventions lasting more than 3 months were found to have a significant impact on reducing anxiety levels.^[26]^

Research indicates that music intervention is an effective treatment for apathy and agitation in dementia, in comparison to other non-pharmacological therapies such as massage, laughter therapy, simulated presence therapy, and dance therapy.^[27]^ This study highlights that passive music was more effective in improving apathy and agitation in older adults. However, in the short term, Holmes et al^[28]^ found that live interactive music was more effective than prerecorded music in reducing apathy in moderate and severe dementia. Additionally, Tsoi et al^[29]^ demonstrated that receptive music intervention was more effective in reducing apathy and agitation in older adults with cognitive impairment, as compared to interactive music intervention. Millan-Calenti et al^[30]^ found that music intervention is an effective non-pharmacological treatment for reducing the agitation of older adults with dementia, especially when the intervention includes personalized and interactive music. Other studies have investigated the efficacy of music intervention on the stress and emotional well-being of older individuals.^[31]^ However, there is a lack of research comparing the effects of short- and long-term music interventions on the mental health of older adults. Therefore, more research is necessary to investigate the impact of music on the health of older adults. In conclusion, the majority of studies show that music intervention has positive or potentially positive effects on the psychological health of older adults, indicating that music intervention can be used as one of the forms of integrative and complementary treatment for the psychological health of older adults.

Music interventions have been shown to improve cognitive function in older adults when compared to nonmusical interventions.^[32]^ Older adults are at higher risk for mild cognitive impairment due to age-related decline in brain function.^[33]^ More than half (52.24%) of the studies included in the evidence map indicate that music interventions are effective in improving the overall cognitive function of older adults. This may be due to the fact that music interventions can enhance the activity of the inferior frontal cortex,^[34]^ and may even be related to changes in the volume of the frontal, temporal, and parietal cortices induced by long-term exposure to music interventions.^[35]^ Additionally, music training has been associated with improvements in memory.^[36]^ Soo Ji Kim et al^[11]^ found that music intervention had a greater impact on the memory and executive function of older adults. Ito et al^[37]^ illustrated that music interventions could improve general cognitive function, executive function, and episodic memory performance in older adults with mild cognitive impairment and dementia. The study also found that group-based music interventions played a relatively better role in improving executive cognition, possibly due to the conducive atmosphere created by active music and group-based activities for older adults. These findings suggest that music interventions could be a valuable non-pharmacological approach to improving cognitive function in older adults, with potential implications for clinical practice.

Music intervention is a non-pharmacological method that has been shown to effectively reduce behavioral and psychological symptoms of dementia.^[38]^ In fact, studies suggest that music intervention is more effective than other non-pharmacological interventions,^[26]^ and it has been found to be effective in treating behavioral and psychological symptoms of dementia.^[39]^ Additionally, physical exercise accompanied by music has been shown to improve the social and behavioral factors of older adults.^[40]^ During the intervention period, positive short-term effects have been observed in the participation, interaction, cognition, and behavior of older adults. Music intervention has also been found to help older adults maintain their attention and regulate their emotions.^[32]^ In general, the influence of music intervention on the cognition of older adults is positive or potentially positive, indicating that music intervention can be considered as an integrative and complementary treatment for cognitive issues in older adults.

In this study, pain, sleep, physical performance, dietary problems, and behavioral symptoms were categorized as physiological aspects of older adults based on the types of studies included. The results demonstrated that music intervention can be effective in improving the physical performance and behavior of older adults. The World Health Organization recommends that older adults engage in at least 150 minutes of moderate-intensity aerobic physical activity per week.^[41]^ However, it has been reported that few older adults meet this standard.^[42]^ Studies have shown that music can be a useful tool to stimulate older adults to increase their participation in physical activities.^[43]^ While few high-quality studies have demonstrated the direct benefits of listening to music during exercise, cumulative benefits can be achieved by incorporating music into physical activity routines over time.^[44]^

Research suggests that music can be an effective intervention for older adults with chronic pain.^[45]^ However, most studies on music intervention are short-term (less than 6 months), and the long-term effects and sustainability of music intervention on pain in older adults have not been fully determined. Furthermore, Liao et al^[46]^ point out that the pain characteristics of most studies are not clear, and the sample size of older adults is small, highlighting the need for larger and more diverse studies with more rigorous research designs. On the other hand, studies have shown that music intervention may help improve sleep quality in older adults, particularly in terms of sleep latency, duration, efficiency, and daytime dysfunction.^[47,48]^ In fact, older adults who listened to music for more than 4 weeks improved their sleep quality more effectively than those who listened to music for less than 4 weeks.^[48]^ This makes music intervention an easy-to-implement and preferred treatment for sleep disorders in older adults, potentially reducing the demand for or dependence on sedatives and sleeping medication. In the map, most of the influences of music intervention on the physiological aspects of older adults are positive or potentially positive, suggesting that music intervention can be considered as one of the forms of integrative and complementary treatment for physiological aspects of older adults.

In the context of the growing aging population, further research is needed to identify effective intervention measures that can improve the health of older adults. One promising approach is the use of music interventions as a health promotion resource, which has demonstrated positive outcomes in the psychology, cognition, and physiology of older adults. Music interventions can also enhance the quality of life, well-being, and satisfaction of older adults. Studies have shown that music interventions can positively impact well-being, subjective health, quality of life, and physical and mental health. Possible mediating factors include strengthening social relations, improving self-confidence and self-esteem, feeling valued, reducing social isolation, and increasing physical activity.^[49]^ Olley and Morales^[27]^ have also shown that music can significantly improve the quality of life, reduce behavioral symptoms, and increase satisfaction among older adults with dementia. Therefore, music interventions may be part of a rational solution to the health problems of older adults. It is essential to adopt a preventive and comprehensive approach to address the psychological problems and chronic diseases of older adults, taking into account the changes in their living environment and lifestyle.

In conclusion, the evidence map indicates that music interventions show promise as integrative and complementary treatments for enhancing psychological health, reducing apathy and agitation, and improving cognitive function in older adults. However, to further enhance the applicability and safety of these interventions, it is essential to consider not only the optimal duration, type, and delivery mode but also carefully assess and define the frequency and intensity. This should be done in conjunction with establishing safety criteria, particularly concerning factors such as the level of agitation, heart rate, and blood pressure, especially when combining music interventions with physical activity. Future research endeavors should aim to address these nuanced aspects for a more comprehensive understanding and application of music interventions across diverse populations of older adults.

## 5. Study limitations

The map only provided a broad overview and was not intended to provide detailed and definitive information on the effectiveness of interventions. Interested researchers should review the identified systematic reviews of interest to obtain a more detailed summary. The purpose of an evidence map was to graphically represent the best evidence found, analyzed, and categorized in order to facilitate access to information for all interested parties. However, it is important to note that this study did not calculate effect sizes for a meta-analysis or assess the risk of bias. we used the AMSTAR 2 tool to critically appraise the quality of the studies included in our evidence map. This enabled us to identify the strengths and weaknesses of each study and provided a more comprehensive and rigorous evaluation of the effects of music interventions on the health outcomes of older adults. It is important to acknowledge that relying on review of reviews may subject the studies to potential publication bias and selection bias. This limitation should be recognized when interpreting the findings and considering the overall strength of the evidence. Future studies should aim to incorporate a more comprehensive analysis of individual randomized controlled trials for each outcome to minimize potential biases. By incorporating the AMSTAR 2 tool, we were able to provide a more robust synthesis of the available evidence and overcome some of the limitations of the evidence mapping approach. Despite these limitations, evidence maps have advantages, as they provide a simple and engaging overview of evidence, which can serve as a useful tool for clinical decision-makers to manage the health of older adults.

## 6. Conclusions

While our evidence map provided a visual overview of the volume and content of music research, it is important to consider how the results can be integrated into clinical practice. Based on our analysis, music interventions have been shown to improve the health outcomes of older adults with various health conditions, including chronic diseases and mental health disorders. This suggests that music could be a safe and effective strategy for implementing health programs for older adults, and may have the potential to reduce the cost and side effects of pharmacological interventions, particularly for conditions such as dementia and mild anxiety and depression.

## Author contributions

**Conceptualization:** Guiyue Ma.

**Formal analysis:** Guiyue Ma.

**Investigation:** Guiyue Ma.

**Methodology:** Guiyue Ma.

**Project administration:** Guiyue Ma.

**Resources:** Guiyue Ma.

**Supervision:** Guiyue Ma.

**Validation:** Guiyue Ma.

**Visualization:** Guiyue Ma.

**Writing – original draft:** Guiyue Ma.

**Writing – review & editing:** Guiyue Ma, Xiaoqin Ma.
